# Calcium amendment for improved germination, plant growth, and leaf photosynthetic electron transport in oat (*Avena sativa*) under NaCl stress

**DOI:** 10.1371/journal.pone.0256529

**Published:** 2021-08-24

**Authors:** Xiaoshan Wang, Qiyue Dingxuan, Mengmeng Shi

**Affiliations:** Department of Grassland Science, College of Animal Science and Technology, Yangzhou University, Yangzhou City, Jiangsu Province, The People’s Republic of China; United Arab Emirates University, UNITED ARAB EMIRATES

## Abstract

Calcium (Ca^2+^) is an essential nutrient element for plants as it stabilizes the membrane system structure and controls enzyme activity. To investigate the effects of Ca^2+^ on plant growth and leaf photosynthetic electron transport in oat (*Avena sativa*) under NaCl stress, oat seeds and plants were cultivated in nutrient solutions with single NaCl treatment and NaCl treatment with CaCl_2_ amendment. By measuring the seed germination rate, plant growth, Na^+^ and Cl^-^ accumulation in leaves, ion leakage in seedlings and leaves, prompt chlorophyll a fluorescence (PF) transient (OJIP), delayed chlorophyll a fluorescence (DF), and modulated 820 nm reflection (MR) values of the leaves at different growth phases, we observed that Ca^2+^ alleviated the inhibition of germination and plant growth and decreased Na^+^ and Cl^-^ accumulation and ion leakage in the leaves under NaCl stress. NaCl stress changed the curves of the OJIP transient, induced PF intensity at P-step (F_P)_ decrease and PF intensity at J-step (F_J_) increase, resulted in obvious K and L bands, and altered the performance index of absorption (PI_ABS_), the absorption of antenna chlorophyll (ABS/RC), electron movement efficiency (ETo/TRo), and potential maximum photosynthetic capacity (F_V_/F_M_) values. With the time extension of NaCl stress, I_1_ and I_2_ in the DF curve showed a decreasing trend, the lowest values of MR/MR_O_ curve increased, and the highest points of the MR/MR_O_ curve decreased. Compared with NaCl treatment, the extent of change induced by NaCl in the values of OJIP, DF and MR was reduced in the NaCl treatment with CaCl_2_ amendment. These results revealed that Ca^2+^ might improve the photosynthetic efficiency and the growth of salt-stressed plants by maintaining the integrity of oxygen-evolving complexes and electron transporters on the side of the PSI receptor and enhancing the relationship between the functional units of the photosynthetic electron transport chain. The findings from this study could be used for improving crop productivity in saline alkali lands.

## Introduction

Soil salinity can seriously affect crop growth at any stage of plant growth, and can ultimately reduce economic yield [1]. Sodium chloride (NaCl), one of the major salts in saline soils, usually causes Na^+^ and Cl^−^toxicity and a low external osmotic potential, inducing the formation of reactive oxygen species (ROS), which could lead to oxidative damage in plants under salinity stress [2,3]. ROS, Na^+^ and Cl^-^, can damage various cell components through oxidative stress and ion toxicity, and can interfere with the normal physiological processes of plant cells [2,3]. The photosynthetic activity of chloroplasts is usually inhibited by NaCl stress, which reduces the photochemical efficiency of photosystem I (PSI) and photosystem II (PSII) [4]. Thus, a better understanding of plant photosynthetic responses to NaCl stress is essential to improve the salt resistance of the plants in saline environments. In recent years, the development of a multi-functional plant efficiency analyzer (M-PEA) has allowed the rapid measurement of the forward and reverse electron transfer and redox states of reaction centers [5–8]. Some photosynthetic electron transfer signals, such as instantaneous fluorescence (PF), delayed fluorescence (DF), and 820 nm modulated reflection (MR), measured synchronously by M-PEA, can reflect the changes in the light energy absorption, transmission, and dissipation, revealing the changes in the photosynthetic processes in stressed plants [9–13].

As light energy is captured by the photochemical reaction center and is transferred through the electron transfer chain, the energy transfer is accompanied by energy dissipation in the form of heat and fluorescence. In the photoelectron transport chain, PF appears after light excitation and before the photochemical reaction uses the excitation energy. The prompt chlorophyll a fluorescence (PF) transient (OJIP) of the PF induction curve is determined by progressive changes in the electronic receptors Q_A_, Q_B,_ and PQ of PSII [9]. DF is produced by electron reverse transfer in the photosynthetic chain. The DF decay curve can reflect multiple emission components in the redox state of PSII [11]. The maximum values of DF at 3 ms (I_1_) and DF at 100 ms (I_2_) are closely related to PSII and PSI after stress, respectively. The MR reflects the redox state of PSI under continuous illumination [5,9]. The absorbance value at 820 nm is directly proportional to the oxidized PSI and plastocyanin (PC) values [9].

Calcium (Ca^2+^) is an essential nutrient element for plants. It stabilizes the membrane system structure and controls enzyme activity [14–16]. When the amount of Ca^2+^ required by plants under normal conditions was applied to salt-stressed plants, salt-stressed plants showed Ca^2+^ deficiency [17]. Adding an appropriate amount of exogenous Ca^2+^ could alleviate Ca^2+^ deficiency, increase the stability and function of the plasma membrane, and ensure the normal transmission of the Ca^2+^ signaling system under salt stress [17–19]. Ca^2+^ is also a necessary factor for photosynthetic oxygen release [16,20], and exogenous Ca^2+^ can significantly increase the chlorophyll content and net photosynthetic rate of some species under salt stress [17,18,21]. However, little information is available on the Ca^2+^-induced changes in the reaction and mechanism of photosynthesis under salt stress.

Oat is a food and forage crop that has great tolerance and adaptability to saline environments, thus, oat has been considered an important crop for improving crop productivity in saline alkali lands. Many studies have been conducted on the gas exchange [22], ion absorption [22–24], and oxidative stress [25–27] of oat grown in saline soils. Under salt stress, the exogenous Ca^2+^ can reduce damage to plants by keeping the stability and functions of the plasma membrane in cells [17–19]. However, there have been few studies on oat salt stress and exogenous Ca^2+^ in photosynthesis, especially on the photosynthetic electron transport during photosynthesis. We hypothesized that salt stress could impair multiple sites in the photosynthetic electron transport chain of oat. Additionally, Ca^2+^ has been reported to play an important role in protecting the photosystems of the electron transport chain from damage caused by salt stress in oat [16–21]. In this study, we measured the seed germination, plant growth, and the signals of PF, DF, and MR in leaves of oat plants under NaCl stress and under NaCl stress with a CaCl_2_ amendment. Our aim was to investigate the effect of NaCl stress on the forward electron flow, backward electron flow, and some electron carriers in the photosynthetic electron transport chain and to reveal the protective mechanism of Ca^2+^ in the photosynthetic electron transport chain.

## Materials and methods

### Oat seed treatment and germination test

Oat seeds were disinfected with 6% NaClO solution for 6 min, washed with sterile water, and dried at room temperature (22–24°C) for 2 h. For each treatment, 50 seeds were placed in each box (11 cm long, 11 cm wide, and 2.5 cm high) with a thin sponge and filter paper placed below to maintain moisture. There were four treatments in this test, including the control (distilled water), 10 mM CaCl_2_, 150 mM NaCl, and 150 mM NaCl + 10 mM CaCl_2_. Each treatment was replicated four times. The seeds were germinated in a light incubator with a photoperiod of 18 h/6 h (day/night) and a temperature regime of 30/25°C (day/night). The water in each box was added every day to ensure consistency in the concentration. On day 7 after beginning the germination test, the germination rate was calculated ((Number of germinated seeds/number of tested seeds) × 100%) and the ion leakage of the seedlings was measured with an electric conductance meter (DDSJ-308A, Scientific Instruments Company, Shanghai, China).

### Controlled pot experiment

Three disinfected oat seeds were planted in each pot (13 cm long, 13 cm wide, and 10 cm high) filled with 1 kg medium quartz sand (0.5 mm) and was watered with 300 mL standard nutrient solution every day. The standard nutrient solution was composed of KNO_3_ (2.5 mM), Ca(NO_3_)_2_ (2.5 mM), (NH_4_)H_2_PO_4_ (0.5 mM), MgSO_4_ 7H_2_O (1 mM), CuSO_4_ 5H_2_O (2×10^−4^ mM), ZnSO_4_ 7H_2_O (1×10^−3^ mM), EDTA.FeNa (0.1 mM), H_3_BO_3_ (2×10^−2^ mM), (NH_4_)_6_Mo_7_O_24_·4H_2_O (5×10^−6^ mM), and MnSO_4_·H_2_O (1×10^−3^ mM). All the plants were placed in a greenhouse with a light (450 μmol m^-2^s^-1^) for 14 h (7:00 am—21:00 pm) every day and with a temperature regime of 28/20°C (light/dark). The nutrient solution was adjusted to pH = 7.0 using 0.01 mM KOH and/or H_2_SO_4_ before solution irrigation. When the plants had three leaves, the 10 mM CaCl_2_, 300 mM NaCl, 300 mM NaCl + 10 mM CaCl_2_, and control (standard nutrient solution with pH = 7) treatments were imposed, with four replications for each treatment.

### Measurements

To determine the electrolytic leakage in the leaves and seedlings, 1.0 g of chopped leaves or seedlings was weighed and soaked in 25 mL deionized water for 24 h. Then, the electric conductivity of the leachates was measured using an electric conductivity meter (DDSJ-308A, Scientific Instruments Company, Shanghai, China).

The electrolytic leakage was calculated using the following formula [28]: electrolytic leakage (%) = [(electric conductivity of treatment–electric conductivity value of control)/electric conductivity value of control] × 100.

After 15 d of NaCl stress, the leaves of each treatment were dried at 105°C for 0.5 h and at 65°C for 48 h to a constant weight, then the leaves were crushed with a miniature crusher. Afterward, 50 mg of the dried sample from each treatment was placed into a 50 mL test tube filled with 20 mL of deionized water. The tubes were placed in boiling water for 1.5 h. After cooling, the extract was filtered into a 25 mL volumetric flask for the determination of Na^+^ and Cl^-^ concentrations using a plasma emission spectrometer (ICP, PE3300DV, Perkin Elmer, Germany) [28].

On the day of NaCl imposition and days of 3, 6, 9, 12, and 15 hereinafter, the plants of each treatment were moved into a dark room. After 3 h in the dark, the 3rd fully expanded leaves of each treatment were used for the measurement of PF, DF, and MR by M-PEA (Hansatech, Norfolk, UK) [29].

The normalized curves in the PF transients were calculated using the following formulas [29]: V_t =_ [(Ft—F_O_)/(F_P_—F_O_)] and Vt = [(Ft—F_O_)/(F_P_—F_O_)].

The curves of the K-band in the PF transients were calculated using the following formulas [29]: W_OJ_ = (Ft—F_O_)/(F_J_—F_O_) and ΔW_OJ_ = W_OJ_^TR^—W_OJ_^0d^.

The curves of the L-band in the PF transients were calculated using the following formulas [29]: W_OK_ = (F_t_—F_O_)/(F_K_—F_O_) and ΔW_OK_ = W_OK_^TR^—W_OK_^0d^.

The parameters, including the performance index of absorption (PI_ABS_), absorption of antenna chlorophyll (ABS/RC), electron movement efficiency (ET_O_/TR_O_), and potential maximum photosynthetic capacity (F_V_/F_M_) were calculated from the OJIP transient [5].

The normalized curves from the DF values were calculated using the following formula [29]: (DF_t_-D_0_)/(DF_I1_-D_0_).

### Statistical analysis

The experiment was a two factorial combination and three treatment: 10 mM CaCl_2_, 150 mM NaCl, and 150 mM NaCl + 10 mM CaCl_2_. All data were collated using Excel 2019 and were subjected to variance analysis using one-way ANOVA (SPSS v11.5). The means of each parameter were separated through Fisher’s least significant difference multiple comparison at the 0.05 probability level.

## Results

**Fig 1:**
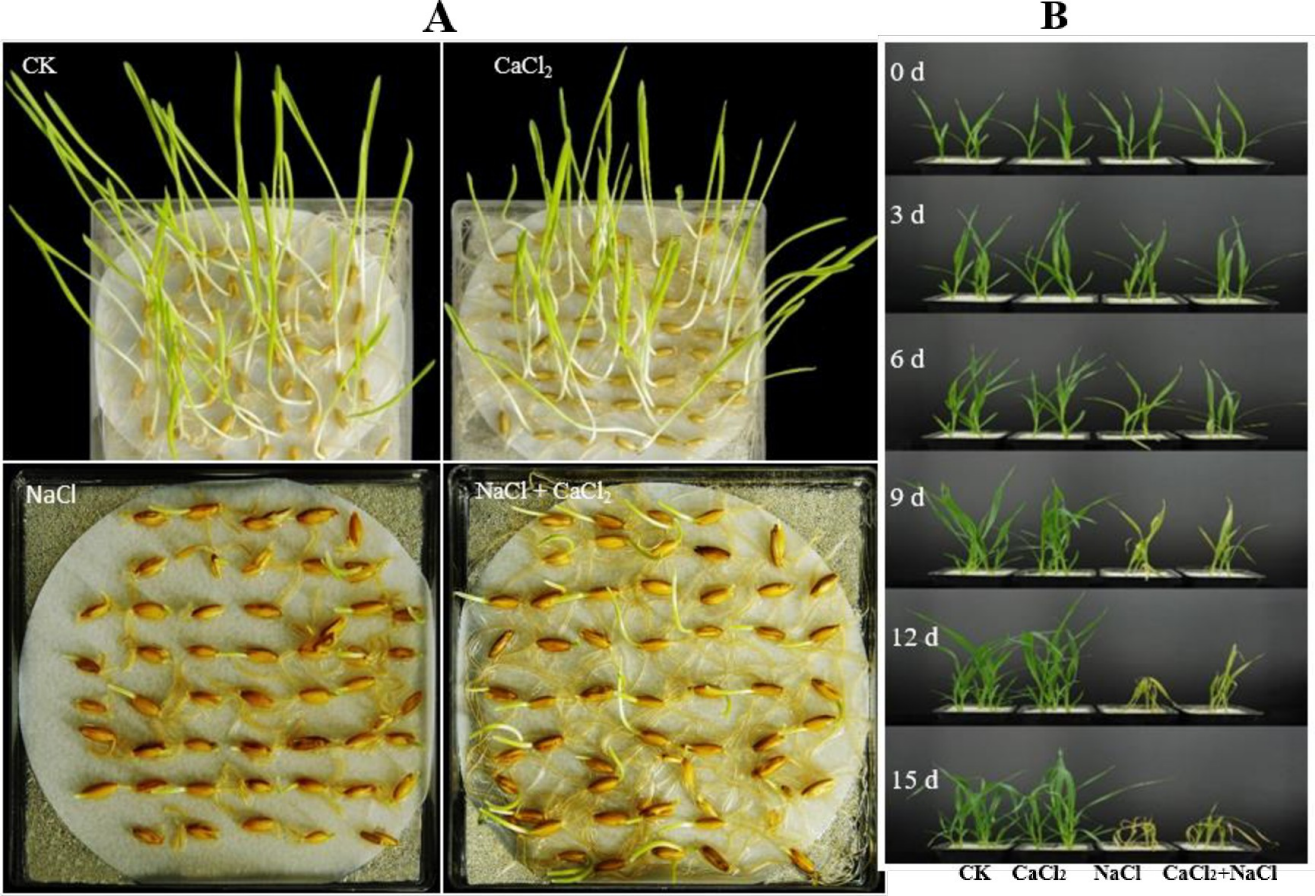
(A) Germination phenotype of oat seeds in the control (water), CaCl_2_ (10 mM), NaCl (150 mM), and NaCl (150 mM) + CaCl_2_ (10 mM) treatments on day 7 day after NaCl stress. (B) The morphological changes of oat plants in the control (nutrient solution), CaCl_2_ (10 mM), NaCl (300 mM), and NaCl (300 mM) + CaCl_2_ (10 mM) treatments on the day of NaCl stress (1) and on days 3 (2), 6 (3), 9 (4), 12 (5), and 15 (6) after NaCl stress. Each treatment of the pictures is the 3 replicates.

The 150 mM NaCl stress significantly inhibited oat seed germination in both the NaCl treatment without CaCl_2_ amendment and the NaCl treatment with CaCl_2_ amendment (Fig 1A and Table 1). Nevertheless, the amendment of Ca^2+^ promoted germination and root extrusion under NaCl stress. Compared with the control, the germination rates decreased by 71% and 44% in the NaCl treatment without CaCl_2_ amendment and NaCl treatment with CaCl_2_ amendment, respectively, on day 7 after NaCl stress (Table 1). Under NaCl stress, the amendment of Ca^2+^ reduced ion leakage of the seedlings (Table 1), increased aboveground dry weight of the plant, and reduced ion leakage of the leaves (Fig 1B and Table 1).

**Table 1:** Germination rate, electrolytic leakages of oat seedlings in the control (water), CaCl_2_ (10 mM), NaCl (150 mM), and NaCl (150 mM) + CaCl_2_ (10 mM) treatments on day 7 after NaCl stress and aboveground dry weight, Na^+^ and Cl^-^ content, and electrolytic leakages of oat leaves in the control (nutrient solution), CaCl_2_ (10 mM), NaCl (300 mM), and NaCl (300 mM) + CaCl_2_ (10 mM) treatments on day 15 after NaCl stress.

Treatment	Germination rates (%)	Electrolytic leakages in seedling (%)	Aboveground dry weight (g plant^-1^)	Na^+^ content (mg g^-1^ DW)		Cl^-^ content (mg g^-1^ DW)	Electrolytic leakage in plants (%)
Control	93±2a	21.2±2.3c	0.51±0.02a	0.05±0.01c	0.02±0.01c		6.1±0.6c
CaCl_2_	92±2a	19.7±1.5c	0.51±0.03a	0.05±0.01c	0.03±0.01c		5.9±0.2c
NaCl	27±5c	52.6±2.3a	0.26±0.01c	1.86±0.14a	0.95±0.09a		22.5±2.6a
NaCl+CaCl_2_	52±6b	40.3±2.0b	0.35±0.03b	1.40±0.16b	0.81±0.02b		18.1±0.2b

Each value is the mean ± SE (n = 3). The data in the same column followed by different letters are statistically different at the 0.05 probability level.

On day 15 after NaCl stress, the leaf Na^+^ and Cl^-^ contents in the NaCl treatment were approximately 37-fold and 48-fold higher than those in the non-NaCl treatment, respectively, whereas the leaf Na^+^ and Cl^-^ contents in the NaCl treatment with CaCl_2_ amendment were approximately 28-fold and 41-fold higher than those in the non-NaCl treatment, respectively (Table 1). These indicated that Ca^2+^ significantly reduced the accumulation of Na^+^ and Cl^-^ in oat leaves under NaCl stress (Table 1).

**Fig 2:**
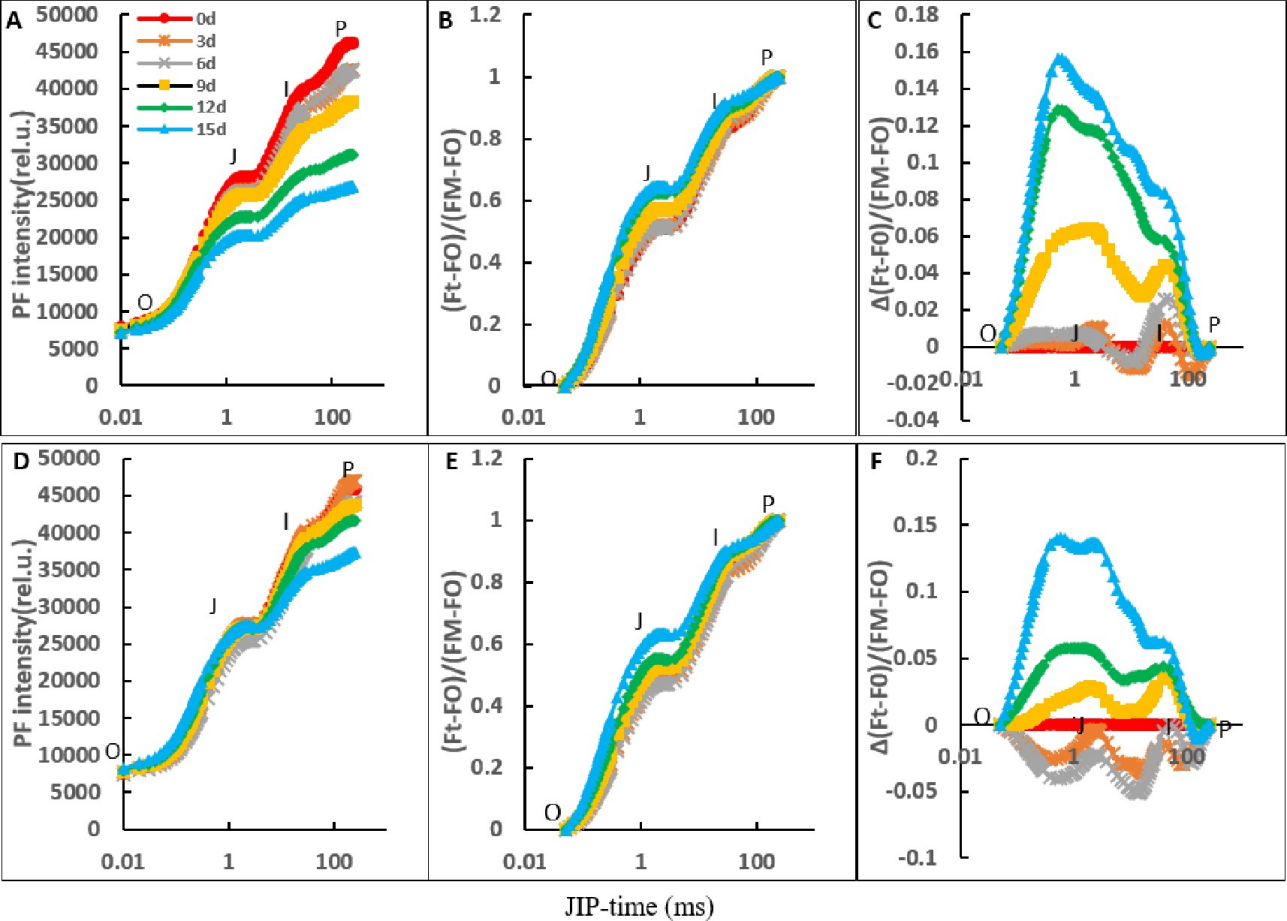
The curves of PF transients of oat leaves in the NaCl treatment and the NaCl treatment with CaCl_2_ amendment on the day of NaCl stress and on days 3, 6, 9, 12, and 15 of NaCl stress. (A, B, C) PF intensity (rel.u.), normalized transients and ΔV_t_ curves in single NaCl treatment. (D, E, F) PF intensity (rel.u.), normalized transients and ΔV_t_ curves in the NaCl treatment with CaCl_2_ amendment. The signals were plotted on a logarithmic time scale (JIP Time). Each value of the curves is the average of 3 replicates.

In the control and CaCl_2_ treatments, leaf PF transients showed a similar shape to the typical polyphasic rise of a basic OJIP. In both NaCl treatments without CaCl_2_ amendment and NaCl treatment with CaCl_2_ amendment, the P value decreased gradually with increasing NaCl stress duration (Fig 2A and 2D). In the NaCl treatment without CaCl_2_ amendment, the P value decreased by 7.9%, 8.3%, 17.3%, 32.6%, and 42.0% on days 3, 6, 9, 12, and 15 of NaCl stress, respectively (Fig 2A). In the NaCl treatment with CaCl_2_ amendment, the P value decreased by 0.1%, 3.9%, 4.9%, 9.4%, and 18.6% on days 3, 6, 9, 12, and 15 of NaCl stress, respectively (Fig 2D).

For the results of transients normalized between O and P, the obvious increase in the J value of OJIP began on day 12 of the NaCl treatment, while in the NaCl treatment with CaCl_2_, the obvious increase in the J value of OJIP began on day 15 (Fig 2B and 2E). In the J phase, the obvious peaks in the ΔVt curves in the NaCl treatment appeared on day 12 after NaCl stress, while in the NaCl treatment with CaCl_2_, obvious peaks in the ΔVt curves appeared on day 15 after NaCl stress (Fig 2B and 2E). For the I point, the obvious peaks in the NaCl treatment appeared on day 3 after NaCl stress, whereas in the NaCl treatment with CaCl_2_ obvious peaks appeared on day 9 of NaCl stress (Fig 2C and 2F).

**Fig 3:**
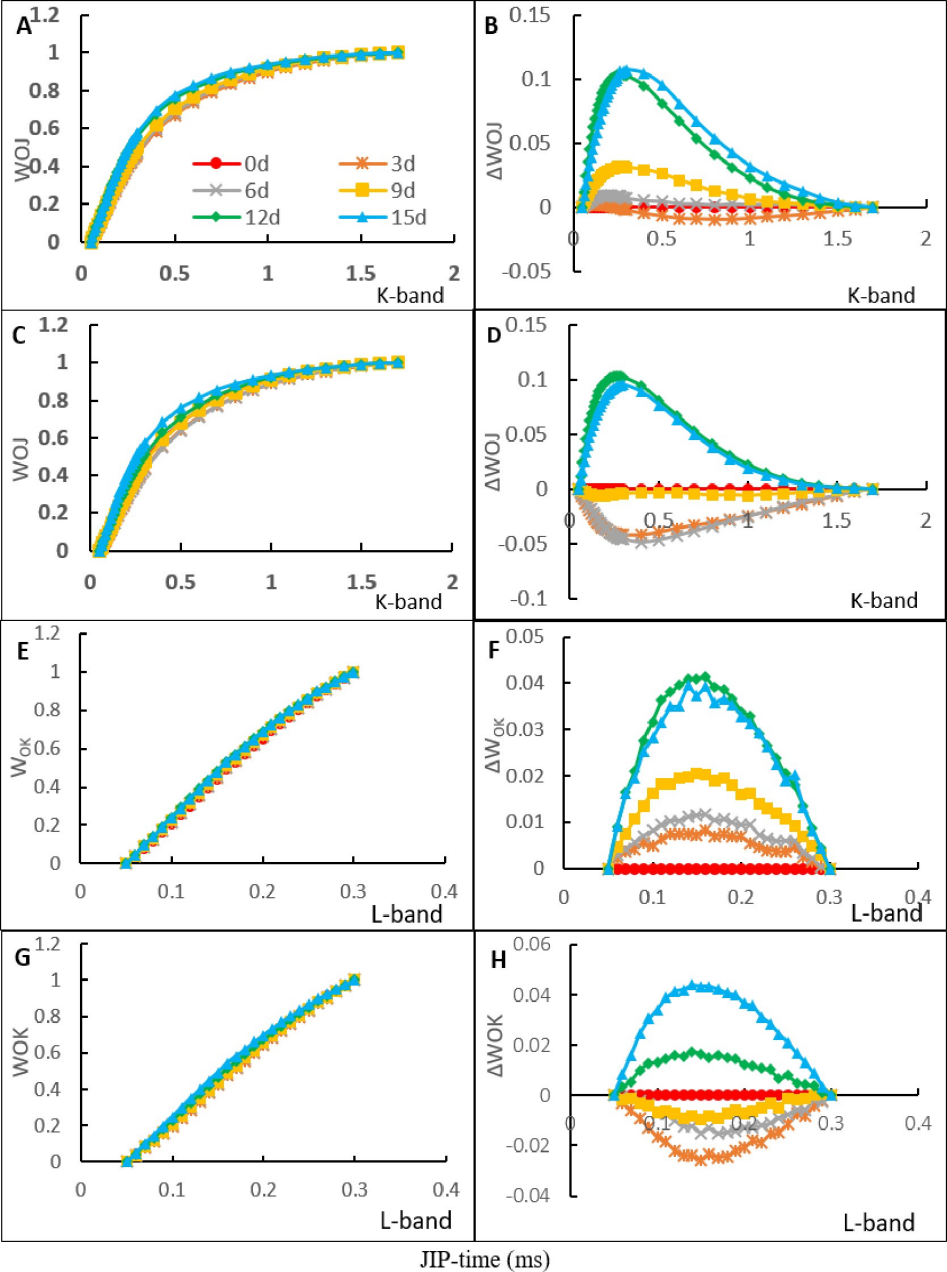
The curves of the K and L band in NaCl treatment and NaCl treatment with CaCl_2_ amendment on the day of NaCl stress and on days 3, 6, 9, 12, and 15 of NaCl stress. (A, B, E, F) the curves of the K-band and L-band in NaCl treatment. (C, D, G, H) the curves of the K-band and L-band in NaCl treatment added CaCl_2_. The signals were plotted on a logarithmic time scale (JIP Time). Each value of the curves is the average of 3 replicates.

With the duration of NaCl stress, the K-band progressively increased in the NaCl treatment and in the NaCl treatment with CaCl_2_ (Fig 3A and 3C). The obvious ascendant K-band in the NaCl treatment was found on day 6 of NaCl stress (Fig 3B). In the NaCl treatment with CaCl_2_, an obvious ascendant K-band was found on day 12 of NaCl stress (Fig 3D). As early as day 3 of NaCl stress, an obvious ascendant L-band appeared in the NaCl treatment (Fig 3F), while in the NaCl treatment with CaCl_2_ amendment, an obvious ascendant L-band appeared on day 12 of NaCl stress (Fig 3H).

**Fig 4:**
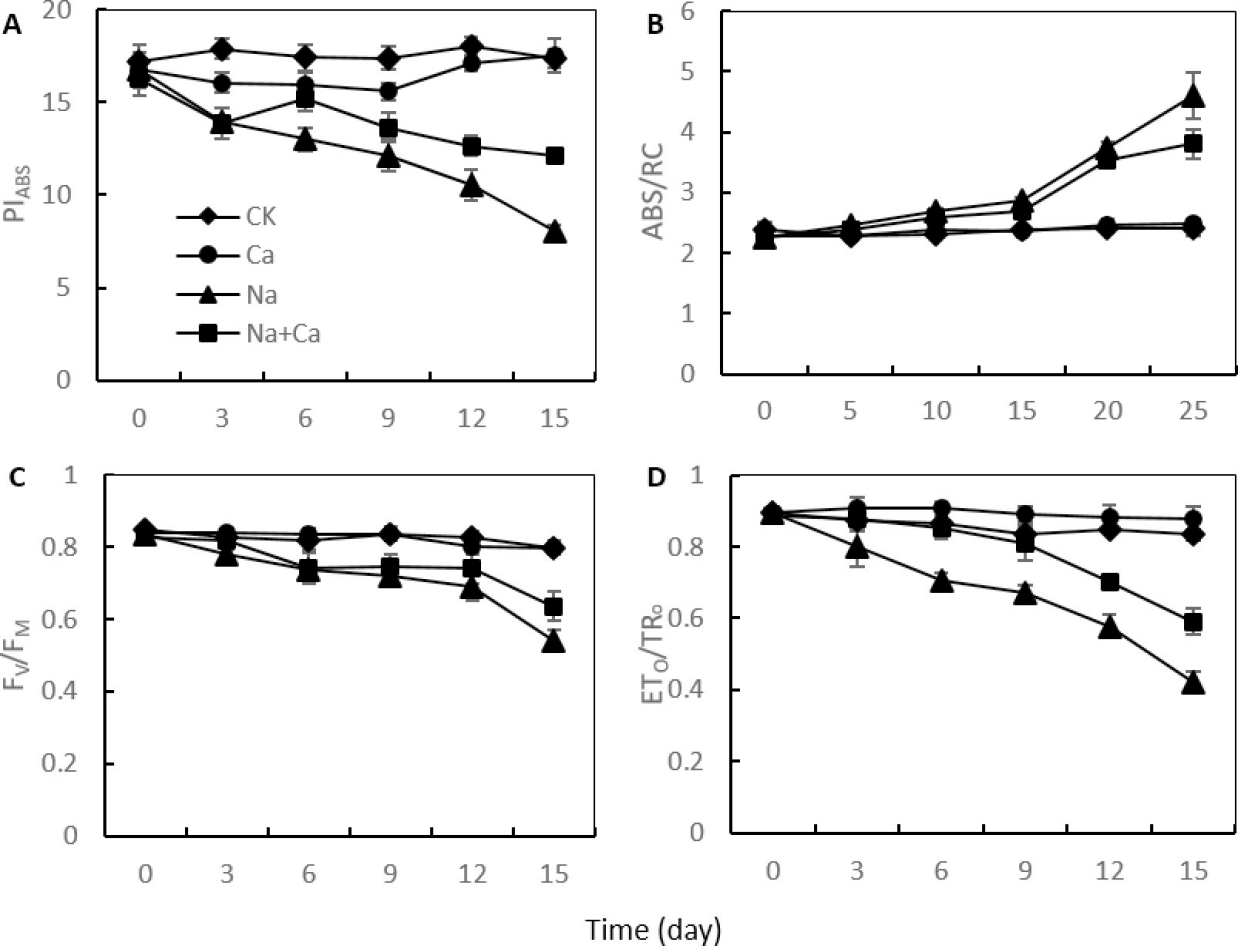
T The parameters of PF OJIP transients of oat leaves in the NaCl treatment and NaCl treatment with CaCl_2_ amendment on the day of NaCl stress and on days 3, 6, 9, 12, and 15 of NaCl stress. (A) PI_ABS_, performance index. (B) ABS/RC, absorption of antenna chlorophyll per PSII reaction center. (C) F_V_/F_M_, potential maximum photosynthetic capacity. (D) ET_O_/TR_O_, efficiency of electron movement. Each parameter is the average of 3 replicates.

For the analysis of the changes in the photosynthetic traits, some parameters (PI_ABS_, ABS/RC, F_V_/F_M_ and ETo/TRo) were calculated from the OJIP transient. The oat leaf values of ABS/RC increased progressively after NaCl stress in both NaCl treatment and NaCl treatment with CaCl_2_ amendment (Fig 4B). In contrast, the PI_ABS_, ETo/TRo and F_V_/F_M_ values decreased progressively (Fig 4A, 4C and 4D). In the NaCl treatment with CaCl_2_ amendment, these parameters showed lesser changes than those in the NaCl treatment, and the changes appeared later with the increase in NaCl stress duration (Fig 4A–4D).

**Fig 5:**
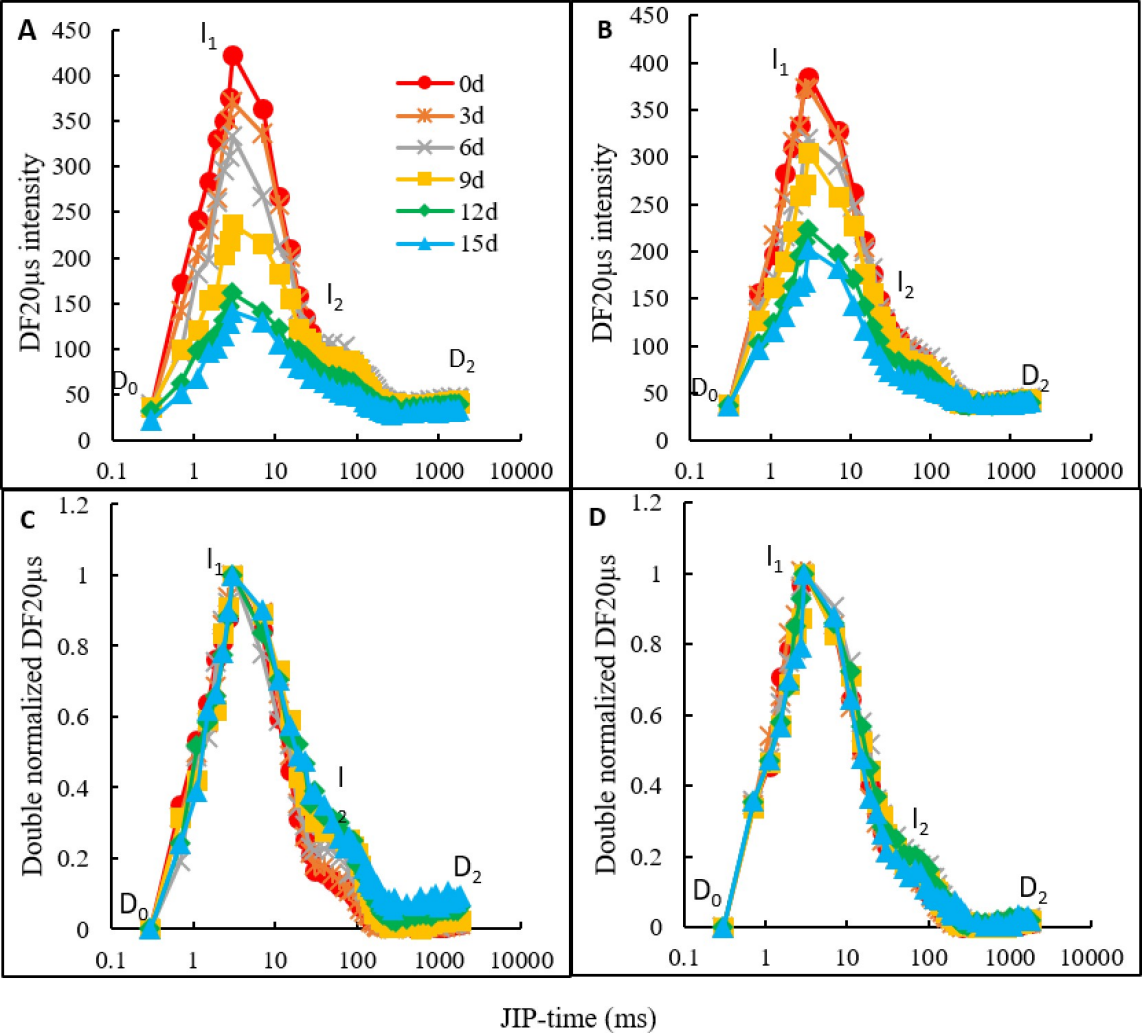
DF induction curves of oat leaves in the NaCl treatment and NaCl treatment with CaCl_2_ amendment on the day of NaCl stress and on days 3, 6, 9, 12, and 15 of NaCl stress. (A, B) The absolute values in NaCl treatment and NaCl treatment added CaCl_2_. (C, D) The normalized curves in the NaCl treatment and NaCl treatment with CaCl_2_ amendment. The D_0_ was the initial value, the peak I_1_ was the value at 3 ms, the I_2_ was the value at 100ms, the D_2_ was the final value of plateau. The signals were plotted on a logarithmic time scale (JIP Time). Each value of the curves is the average of 3 replicates.

For the plants, a DF induction curve was constructed for the signals measured at 20ms-delay per dark interval, which increased between the initial D_0_ and I_1_ in 3ms, then decreased to the platform D_2_, and passed through the I_2_ in 100ms. In both the NaCl treatment and the NaCl treatment with CaCl_2_ amendment, the amplitude and shape of the DF induction curves were changed by NaCl stress (Fig 5A and 5B). NaCl stress led to a progressively decreased DF amplitude, and the reduction was more notable at the I_1_ than at I_2_ (Fig 5A and 5B). The DF amplitude in the NaCl treatment showed a much faster decrease than that in the NaCl treatment with CaCl_2_ amendment (Fig 5A and 5B).

In the NaCl treatment, the I_1_ maximum decreased by 12.4%, 21.2%, 44.1%, 62.0%, and 66.6% on days 3, 6, 9, 12, and 15 of NaCl stress, respectively (Fig 5A). In the NaCl treatment with CaCl_2_ amendment, the I_1_ maximum decreased by 3.5%, 17.2%, 21.0%, 42.2%, and 47.4% on days 3, 6, 9, 12, and 15 of NaCl stress, respectively (Fig 5B). In the normalization curves of both D_0_ and I_1_, the normalized I_2_ values in the NaCl treatment were increased by NaCl stress (Fig 5C), while in the NaCl treatment with CaCl_2_ amendment, there was no obvious change in the normalized I_2_ values (Fig 5D). The I_2_/I_1_ ratio in the NaCl treatment increased, while in the NaCl treatment with CaCl_2_ amendment, little change was observed (Fig 5C and 5D).

**Fig 6:**
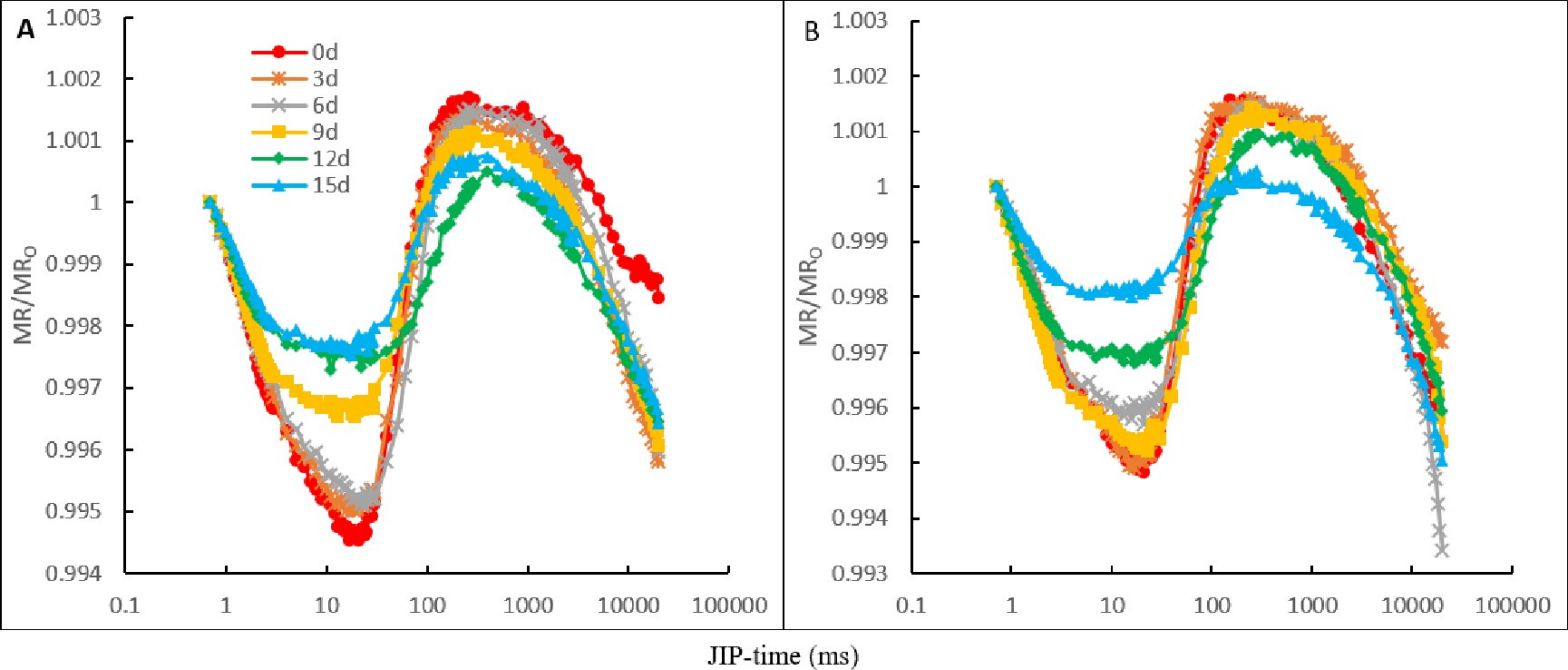
The modulated 820-nm reflection kinetics (MR/MR_O_) of oat leaves in NaCl treatment and NaCl treatment with CaCl_2_ amendment on the day of NaCl stress and on days 3, 6, 9, 12, and 15 of NaCl stress. (A)The MR/MR_O_ curves in the NaCl treatment. (B) The MR/MR_O_ curves in NaCl treatment added CaCl_2_. The signals were plotted on a logarithmic time scale (JIP Time). Each value of the curves is the average of 3 replicates.

In the control and CaCl_2_ treatments, the curves of the MR/MR_O_ at 820 nm in the oat leaves showed a pattern of decline first, which then increased. In the NaCl treatment and the NaCl treatment with CaCl_2_ amendment, the curve pattern of the MR/MR_O_ changed significantly (Fig 6A and 6B). The NaCl stress induced an elevation in the descending phase of the MR/MR_O_ curves and a reduction in the ascending phase of the MR/MR_O_ curves in both the NaCl treatment and the NaCl treatment with CaCl_2_ amendment. On day 9 of NaCl stress, the decrease then increase curve pattern of the MR/MR_O_ was eliminated partially in the NaCl treatment (Fig 6A), whereas in the NaCl treatment with CaCl_2_, on day 12 of NaCl stress (Fig 6B).

## Discussion

Salinity stress can cause damage to cellular membrane structure and can interfere with cellular functions at any stage of the plant growth [2,3,30]. In salt treatment, Ca^2+^ amendment can alleviate damage to plants by keeping the stability and functions of the plasma membrane in cells [17,18]. In the present study, the growth of salt-stressed oat seedlings and plants was improved by the addition of Ca^2+^. The lower ion leakage of oat seedlings and leaves in the NaCl treatment with CaCl_2_ amendment than in NaCl treatment supported the idea that Ca^2+^ could control membrane structure and function of cells by blocking Na^+^ influx, binding with phospholipids, and stabilizing lipid bilayers [16], thus, alleviating the effects of NaCl stress on plants by controlling membrane permeability [17–19]. The components of the photosynthetic electron transfer chain are located on the thylakoid membrane in plant cells. Salt stress damages the structure of the thylakoids in chloroplasts of leaves and decreases photosynthetic pigments contents and photosynthetic efficiency in some plant species [4,29,31–36]. Exploring the effects of Ca^2+^ on the photosynthetic electron transfer under salt stress could further reveal the possible mechanisms of Ca^2+^-enhanced salt tolerance of oats.

Changes in the OJIP transients of PF in oat leaves revealed that the normal photosynthetic electron transport is affected by salt stress in both NaCl treatment and NaCl treatment with CaCl_2_ amendment. As NaCl stress continued, decreased I-P stage in OJIP curves, increased J-I stage in OJIP curves, and changed K-band and L-band were observed. Similar results have also been found in other plant studies [29,34]. The decreased I-P stage in OJIP under continuous NaCl stress revealed that salt stress reduced the redox capability of PSI [8,9]. The decreased P value was caused by degradation and denaturation of chlorophyll proteins at the PSI acceptor side and decreased numbers of closed PSII RCs [7,37]. The increased J-I stage in normalized transients of OJIP under continuous NaCl stress suggested that the active PQ molecular stock was reduced by salt stress in PSII [20]. At the later stage of NaCl stress, the higher P value and amplitude of the I-P phase in the OJIP curves in NaCl treatment with CaCl_2_ amendment than in NaCl treatment indicated that Ca^2+^ inhibited the degradation and denaturation of chlorophyll proteins at the PSI acceptor side and improved the redox rate of PSI under NaCl stress. The increased J point in normalized transients of OJIP suggested that the transfer rate of electrons from P_680_ to Q_A_ was limited and the quantum yield of PF increased [37]. The changes in the K-band and L-band in the present study indicated that the oxygen-evolving complex was impaired by NaCl stress. Moreover, NaCl stress caused a reduction in electron transfer efficiency in the PSII donor side and inhibited the energy flow of PSII [12,20]. The obvious differences in the K-band and L-band between the NaCl treatment and NaCl treatment with CaCl_2_ amendment suggested that Ca^2+^ increased the electron transfer efficiency in the PSII donor side by protecting the oxygen-evolving complex under NaCl stress.

Several parameters, such as PI_ABS_, ABS/RC, ETo/TRo and F_V_/F_M_ developed from the OJIP transient, can provide detailed the information on the photosynthetic process [38,39]. In the present study, the increase in ABS/RC and the decrease in PI_ABS_ indicated that NaCl stress reduced the number of active PSII reaction centers and decreased the overall PSII activity of oat leaves in NaCl treatment and NaCl treatment with CaCl_2_ amendment. The reduced number of active PSII reaction centers could lead to an increase in the necessary number of RC turnovers for full reduction of the PQ pool [20]. Compared with NaCl treatment, the changes in NaCl stress on ABS/RC and PI_ABS_ in oat leaves showed a lesser extent and appeared at a later stage in NaCl treatment with CaCl_2_ amendment. The results indicated that Ca^2+^ could reduce the damage of NaCl stress on PSII reaction centers in the leaves of oat. The decrease in ET_O_/TR_O_ and F_V_/F_M_ in oat leaves suggested that NaCl stress decreased the capability of PSII to capture light energy for Q_A_ reduction in both NaCl treatments. Similarly, previous studies in maize have shown that salt stress decreases the open PSII energy capture efficiency [29]. The changes in NaCl stress on ET_O_/TR_O_ and F_V_/F_M_ in oat leaves were to a lesser extent and appeared at a later stage in the NaCl treatment with CaCl_2_ amendment. The results indicated that Ca^2+^ improved the capability of PSII to capture light energy for Q_A_ reduction under NaCl stress.

NaCl stress decreased the intensity of DF and made the speed of reaching I_1_ relatively faster in both the NaCl treatment and NaCl treatment with CaCl_2_ amendment. The results indicated that NaCl stress decreased the activation of RCs, resulting in weakened backward electron transfer capacity and electron refilling of chlorophyll in the excited PSII antenna [5]. The I_1_ value of the DF curves was affected by the transmembrane electrical gradient and Z^+^P_680_Q_A_Q_B_^-^ accumulation in reaction centers, and the I_2_ peak of DF was related to the reduction of PQ and the Z^+^P_680_Q_A_^-^Q_B_^-^ increase in reaction centers [7,40]. The decreased I_1_ and I_2_ values suggested that the electron transfer capacity in both the donor and acceptor sides of PSII decreased due to NaCl stress [6,11]. At the later stage of NaCl stress, the higher amplitudes at I_1_ and I_2_ phases in the DF curves in the NaCl treatment with CaCl_2_ amendment than that in NaCl treatment suggested that Ca^2+^ relieved the salt injuries to the donor and acceptor sides of PSII and improved the transmembrane electrical gradient and backward electron transfer capacity in the oat leaves under NaCl stress.

The lowest point of the decline phase of the MR/MR_O_ transient significantly increased at the later stage of NaCl stress in both the NaCl and NaCl treatments with CaCl_2_ amendment. This result showed that NaCl stress reduced the oxidation activity of PSI. In the NaCl treatment and NaCl treatment with CaCl_2_ amendment, the signal weakening of the MR/MR_O_ transient in the duration of NaCl stress showed that NaCl stress reduced the reduction activity of PSI. The reduction in the activity of PSI may be due to the decrease in the capacity of PSII to produce and transfer electrons, the electron transfer obstruction between PSII and PSI, and the inhibition of the receptor side of PSI [35]. Combining the results of the OJIP transients with their parameters and DF parameters, it can be deduced that NaCl stress damaged multiple sites of the photosynthetic electron transport chain [29]. Under the NaCl treatment, the lowest point of decline in the MR/MR_O_ transient emerged earlier than that of the NaCl treatment with CaCl_2_ amendment. These results showed that Ca^2+^ alleviated the damage on PSII and on some sites of the photosynthetic electron transport chain under NaCl stress.

## Conclusions

In the present study, according to the increase in the ion leakage in the oat seedlings and leaves, we concluded that the 150 mM NaCl treatment damaged the cells in the oat seedlings, and the 300 mM NaCl treatment damaged the cells in the leaves of oat plants. The addition of 10 mM CaCl_2_ improved seed germination and plant growth, decreased the accumulation of Na^+^ and Cl^-^ in the leaves of plants, and reduced the ion leakage of the oat seedlings and plant leaves. The changed curves of the PF, DF, and MR in the oat leaves at plant growth phases revealed that NaCl stress interfered with the oat photoreaction system in the NaCl treatment and NaCl treatment with CaCl_2_ amendment. The oxygen-evolving complexes and electron transporters of the oat leaves were damaged by NaCl stress on the PSI receptor side. The NaCl treatment reduced the activity of the PSII reaction center and the interlinkage between functional units in the photosynthetic electron transport chain and decreased the rate of electron flow from Q_B_ to Q_A_ in the oat leaves. Compared with the single NaCl treatment, the degree of change in the photosynthetic electron transport induced by NaCl was decreased by the addition of Ca^2+^ in the NaCl treatment with CaCl_2_ amendment. These results showed that Ca^2+^ could alleviate the damages caused by NaCl stress to the photosynthetic electron transporters and improve the salt resistance of plants. Further research on Ca^2+^ fertilizers of soil could improve crop yield in saline-alkali soil.

## Supporting information

S1 File (ZIP)

## Abbreviations

ABS/RC: the absorption of antenna chlorophylls (ABS) per PSII reaction center (RC)
DF: delayed chlorophyll a fluorescence
ETO/TRO: probability that an electron moves further than QA-
F_I_: PF intensity at I-step
F_J_: PF intensity at J-step
Fo: PF intensity at O- step
Fp: PF intensity at P-step
FV/FM: the potential maximum photosynthetic capacity
M-PEA: Multi-functional Plant Efficiency Analyzer
MR: modulated 820nm reflection
OEC: oxygen evolving complex
OJIP: prompt chlorophyll a fluorescence transient
PC: plastocyanin
PF: prompt chlorophyll a fluorescence
PQ: plastoquinone
PSI: photosystem I
PSII: photosystem II
PSs: the photochemical efficiency of two photosystems
QA: the primary quinone electron acceptor of PSII
QB: the secondary quinone electron acceptor of PSII
ROS: reactive oxygen species
VPSI: maximum slope decrease of MR/MRO
VPSII-PSI: maximum slope increase of MR/MRO
WOJ: the ratio of variable fluorescence Ft -FO to the amplitude FJ -FO
WOK: the ratio of variable fluorescence Ft -FO to the amplitude FK -FO
