# Supplementary material for: Calcium amendment for improved germination, plant growth, and leaf photosynthetic electron transport in oat (Avena sativa) under NaCl stress
Source: PLoS One. 2021 Aug 24;16(8):e0256529. doi: 10.1371/journal.pone.0256529 (PMC8384207; doi:10.1371/journal.pone.0256529)
Supplement: S1 File — (ZIP) [file pone.0256529.s001.zip › Morphological changes of seedlings and plants.doc]

**Figure 1**. (A) Germination phenotype of oat seeds in the control (water), CaCl_2_ (10 mM), NaCl (150 mM), and NaCl (150 mM) + CaCl_2_ (10 mM) treatments on day 7 day after NaCl stress. (B) The morphological changes of oat plants in the control (nutrient solution), CaCl_2_ (10 mM), NaCl (300 mM), and NaCl (300 mM) + CaCl_2_ (10 mM) treatments on the day of NaCl stress (1) and on days 3 (2), 6 (3), 9 (4), 12 (5), and 15 (6) after NaCl stress. Each treatment of the pictures is the 3 replicates.
